# RAN Translation-Coupled Decay of the *C9orf72* GGGGCC Repeat Transcript by the RNA Exosome Suppresses Dipeptide Repeat Production

**DOI:** 10.3390/ijms27156986

**Published:** 2026-08-04

**Authors:** You Wu, Li Li, Jing Tian, Leilei Liu, Kunzhao Du, Zhicheng Shao, Tianlin Cheng, Xin Cao, Tao Wang

**Affiliations:** 1 Institute of Clinical Sciences, Zhongshan Hospital, Fudan University, Shanghai 200032, China; wuyou_9602@163.com (Y.W.); 23111530005@m.fudan.edu.cn (L.L.); 22111520040@m.fudan.edu.cn (J.T.); liuleilei0330@163.com (L.L.); dukunzhao@fudan.edu.cn (K.D.); 2 Institute for Translational Brain Research, State Key Laboratory of Brain Function and Disorders, MOE Frontiers Center for Brain Science, Fudan University, Shanghai 200031, China; zcshao@fudan.edu.cn (Z.S.); chengtianlin@fudan.edu.cn (T.C.); 3 Department of Neurology, Zhongshan Hospital, Fudan University, Shanghai 200032, China; 4 Institute of Pediatrics, Children’s Hospital, Fudan University, Shanghai 200031, China

**Keywords:** RAN translation, C9orf72-ALS, RNA exosome, co-translational regulation, mRNA stability

## Abstract

The RNA exosome plays a critical role in surveilling nuclear mRNA biogenesis and regulating co-translational mRNA decay in the cytoplasm. Unlike canonical translation, repeat-associated non-AUG (RAN) translation of a GGGGCC hexanucleotide repeat expansion (HRE) within an intron of the *C9orf72* locus leads to the synthesis of neurotoxic dipeptide-repeat (DPR) proteins, contributing to the pathogenesis of frontotemporal dementia and amyotrophic lateral sclerosis (C9-ALS/FTD). However, it remains unclear whether aberrant RAN translation is monitored and regulated co-translationally or how *C9orf72* HRE (C9-HRE) mRNA is degraded during this process. Here, we demonstrate that RAN translation triggers the rapid decay of C9-HRE mRNA. During this process, the RNA exosome engages the translating ribosome-C9-HRE mRNA complex to mediate RAN translation-coupled mRNA decay. Moreover, overexpression of EXOSC3, a key subunit of the RNA exosome cap, promotes RAN translation-coupled decay of C9-HRE mRNA and suppresses DPR production. In iPSC-derived neurons, a reduction in EXOSC3 levels blocks C9-HRE mRNA decay in a translation-dependent manner, further confirming its role in RAN translation surveillance. These findings highlight the essential function of the RNA exosome, particularly EXOSC3, in mitigating RAN translation-associated toxicity and preventing pathological DPR production. This work provides insights into potential therapeutic strategies for C9-ALS/FTD and may have broader implications for other disorders involving RAN translation.

## 1. Introduction

Cells maintain accurate translation to ensure proper protein synthesis. Canonical translation initiates when the small (40S) ribosomal subunit scans the mRNA to recognize the AUG start codon, followed by the joining of the large (60S) subunit [1,2]. During this process, co-translational mRNA decay serves as an indispensable quality control mechanism that safeguards cellular protein homeostasis (proteostasis) [3,4,5]. Aberrant mRNAs, such as those lacking stop codons, harboring premature termination codons, or containing stable secondary structures, can induce ribosome stalling and activate specialized mRNA surveillance pathways, including nonsense-mediated decay (NMD), non-stop decay (NSD), and no-go decay (NGD) [5,6]. This surveillance is particularly critical in post-mitotic neurons due to their heightened sensitivity to proteotoxic stress. Consequently, dysregulation of co-translational decay and ribosome stalling resolution is increasingly implicated as a core driver of neurodegenerative diseases, including amyotrophic lateral sclerosis (ALS) and frontotemporal dementia (FTD) [7,8,9,10,11]. Failure to efficiently eliminate aberrant mRNAs and stalled ribosome complexes results in the accumulation of neurotoxic proteins and misfolded aggregates [10,12].

While eukaryotic mRNA translation typically begins at the AUG start codon on transcripts bearing a 5′ cap structure, nucleotide repeat expansions, which cause more than 50 neurological diseases, can drive translation in the absence of AUG start codons via both cap-dependent and cap-independent mechanisms [13,14,15,16,17]. This phenomenon, termed repeat-associated non-AUG (RAN) translation, is exemplified by the GGGGCC hexanucleotide repeat expansion (HRE) in *C9orf72*, the most common genetic cause of amyotrophic lateral sclerosis (ALS) and frontotemporal dementia (FTD) [18,19]. RAN translation occurs in all three reading frames of C9-HRE, generating three dipeptide repeat (DPR) proteins: poly-GA, poly-GP, and poly-GR. These DPRs are highly neurotoxic, and their accumulation is a pathological hallmark of C9-ALS/FTD [20,21,22,23,24]. Moreover, DPR expression alone is sufficient to recapitulate ALS/FTD pathological and behavioral phenotypes in multiple in vivo models [23,25,26,27,28,29]. Consistently, suppression of RAN translation using gene editing, without altering the C9-HRE sequence or expression levels, fully rescues motor and cognitive behavioral defects in mice [30]. Thus, RAN translation activity dictates DPR production and drives the subsequent pathogenesis of C9-ALS/FTD.

Recent studies have shown that RAN translation induces ribosome stalling, partly due to stable RNA secondary structures and electrostatic interactions between positively charged arginine residues in poly-GR/poly-GP and the ribosomal exit tunnel [31,32,33]. Moreover, multiple ribosomal quality control factors, including ZNF598, NEMF, LTN1, and ANKZF1, promote the degradation of nascent DPR chains [3,34,35,36,37]. Consequently, the depletion of these factors exacerbates RAN product accumulation, indicating that aberrant RAN translation is subject to active surveillance mechanisms that limit DPR production and mitigate disease progression. However, the mechanisms by which C9-HRE-containing mRNAs are monitored during RAN translation remain largely unknown.

The evolutionarily conserved RNA exosome mediates 3′-to-5′ RNA degradation in eukaryotes [38,39]. It participates in mRNA surveillance in the cytoplasm and performs diverse functions in the nucleus, ranging from elimination of defective RNAs to the processing of structured RNA species. Structurally, the human RNA exosome consists of a catalytically inactive, barrel-shaped core composed of nine subunits (EXOSC1-9): six PH-like proteins that form a pseudo-hexameric ring, and three proteins with S1/KH domains that form a cap [38,40]. This core serves as a scaffold that channels RNA substrates toward catalytic subunits. The exosome’s nucleolytic activity is conferred by associated hydrolytic enzymes, primarily DIS3 and DIS3L, which localize predominantly to the nucleus and cytoplasm, respectively [41]. In addition, the nuclear exosome contains the catalytic subunit EXOSC10, which further contributes to 3′-to-5′ exoribonuclease activity [41].

In the cytoplasm, the RNA exosome plays a pivotal role in co-translational mRNA decay pathways [42]. For example, during NSD, the cytoplasmic RNA exosome is recruited to ribosomes stalled at the 3′ poly(A) tail, executing rapid 3′-to-5′ degradation of aberrant transcripts that lack termination codons [6]. During NGD, following endonucleolytic cleavage of mRNAs at stalled ribosomes, the RNA exosome is responsible for clearing the resulting upstream 5′ cleavage product via its 3′-to-5′ exoribonuclease activity [43]. While it has been reported that the nuclear RNA exosome can degrade the C9-HRE transcript, thereby indirectly limiting the production of neurotoxic DPRs [44], it remains unclear whether the cytoplasmic RNA exosome directly participates in co-translational surveillance of RAN translation.

In this study, we identify an RNA exosome-mediated co-translational surveillance mechanism that targets RAN translation in the cytoplasm. We demonstrate that this pathway promotes rapid degradation of C9-HRE mRNAs and plays a critical role in suppressing DPR production.

## 2. Results

### 2.1. Co-Translational Degradation of GGGGCC mRNA as a Quality Control Mechanism

**Figure 1:**
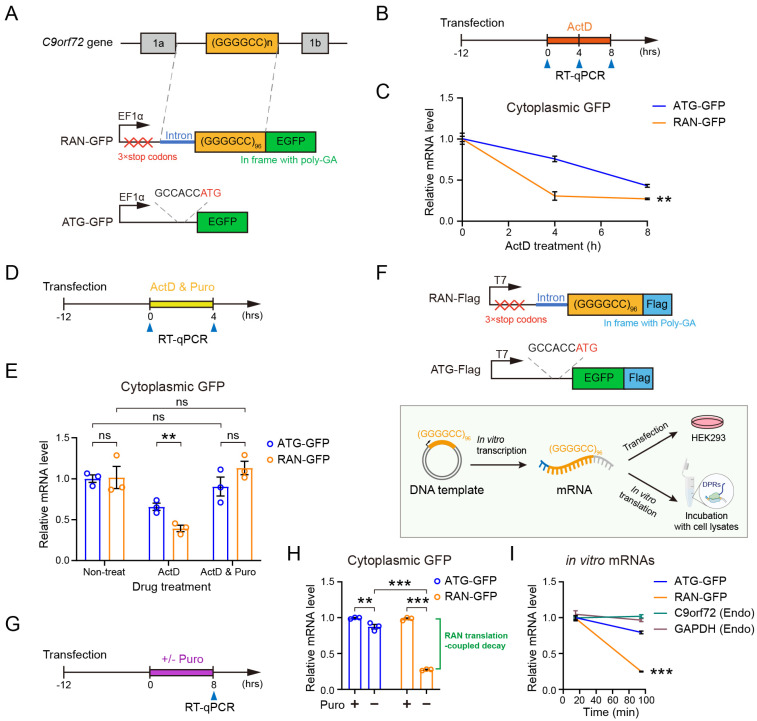
Co-translational degradation of GGGGCC mRNA. (**A**) Schematic representation of the human C9-HRE transcript and the RAN translation reporter (RAN-GFP), which contains 96 GGGGCC repeats and an upstream intronic sequence driven by the EF1α promoter. Multiple stop codons in all three reading frames were inserted 5′ to the intron to prevent potential canonical translation. A parallel GFP reporter (ATG-GFP) was constructed to serve as a canonical translation control. Detailed reporter sequences are provided in Table S1. (**B**,**C**) Treatment scheme: cells were transfected with the indicated plasmids for 12 h, followed by an 8 h treatment with actinomycin D (ActD). Cells were harvested at the indicated time points for subcellular fractionation. Cytoplasmic reporter mRNA abundances were analyzed by reverse transcription quantitative PCR (RT-qPCR) (n = 3). (**D**,**E**) Treatment scheme: cells were transfected with the indicated plasmids for 12 h, followed by a 4 h treatment with ActD in the presence or absence of puromycin (Puro). Cells were harvested at the indicated time points, and cytoplasmic reporter mRNA abundances were analyzed by RT-qPCR (n = 3). (**F**) Scheme of the RAN-GFP and ATG-GFP reporters driven by a T7 promoter. Constructs were transcribed in vitro using T7 RNA polymerase, capped, and subsequently either transfected into HEK293T cells or used for in vitro translation with soluble HEK293T cell lysates. Detailed reporter sequences are provided in Table S1. (**G**,**H**) Treatment scheme: cells were transfected with the indicated in vitro-transcribed mRNA for 12 h, with or without subsequent puromycin treatment for 8 h. Cells were harvested 20 h post-transfection, and cytoplasmic reporter mRNA abundances were analyzed by RT-qPCR (n = 3). (**I**) Reporter mRNA abundances in the in vitro translation mixtures 15 and 90 min after the addition of translation buffer to initiate translation. The mRNA abundances of endogenous (Endo) *C9orf72* and *GAPDH* in the mixtures were also analyzed (n = 3). Data are presented as mean ± SEM. Statistical significance was determined using two-way ANOVA for (**C**,**I**) and unpaired two-sided Student’s *t*-test for (**E**,**H**); ** *p* < 0.01; *** *p* < 0.001; ns, not significant.

To investigate the RAN translation of C9-HRE mRNA, we constructed a GFP reporter based on previously established strategies to monitor RAN translation activity [45,46,47]. This reporter contains 96 GGGGCC repeats preceded by the upstream intronic sequence of human *C9orf72*, followed by GFP in-frame with the poly-GA reading frame (Figure 1A and Table S1, RAN-GFP). We also constructed a corresponding GFP reporter to assess ATG-dependent canonical translation (Figure 1A and Table S1, ATG-GFP). Forty-eight hours after transfecting each reporter into HEK293T cells, Western blot analysis using anti-GP and anti-GA antibodies revealed robust DPR production specifically in the C9-HRE group (Figure S1A). Fluorescence imaging further revealed that GFP signals in the C9-HRE group predominantly formed highly compact cytoplasmic inclusions, consistent with the known distribution pattern of poly-GA (Figure S1B). These findings demonstrate that the C9-HRE reporter reliably captures RAN translation activity and can be utilized to study its regulation.

We next assessed the stability of the C9-HRE mRNA undergoing RAN translation compared to GFP mRNA undergoing conventional translation. Following the transfection of equal amounts of both reporter plasmids, cells were treated with Actinomycin D (ActD), a transcription inhibitor used to evaluate mRNA stability (Figure 1B). We observed that C9-HRE mRNA decayed faster than GFP mRNA (Figure 1C). To determine whether this accelerated decay was dependent on translation, cells were treated with ActD in the presence of puromycin, which incorporates into nascent polypeptides, causing translation termination, ribosome disassembly, and mRNA release. Puromycin treatment completely blocked the decay of both C9-HRE and GFP mRNAs, resulting in comparable mRNA levels (Figure 1D,E). These findings suggest that RAN translation triggers faster C9-HRE mRNA decay.

Because aberrant mRNA can also be degraded during nuclear transcription and processing, we sought to exclude the possibility that the reduced stability of C9-HRE mRNA arises from the inherent structural instability affecting its initial transcription and production. To this end, we synthesized the C9-HRE mRNA and control GFP mRNA via in vitro transcription (Figure 1F and Table S1). The purity and integrity of the synthetic transcripts were confirmed by agarose gel electrophoresis (Figure S1C). Upon transfecting these synthetic mRNAs into cells, we observed GFP-positive cytoplasmic poly-GA inclusions in the C9-HRE group, confirming successful RAN translation of the synthetic transcripts (Figure S1D). We then assessed the rate of translation-dependent mRNA decay using puromycin to isolate this pathway from translation-independent degradation mechanisms (Figure 1G). The reduction in the ratio of untreated cells (−puro) to puromycin-treated cells (+puro) indicated the extent of mRNA degradation during translation. Consistent with our previous results, the translation-associated decay of C9-HRE mRNA was faster than that of the ATG-GFP control (Figure 1H).

Finally, we established a cell lysate-based in vitro translation system to further dissect the RAN translation-coupled decay of C9-HRE mRNA (Figure 1F). Incubation of the synthetic control GFP or C9-HRE mRNA with translation buffer and cytosolic soluble lysate derived from wild-type HEK293T cells resulted in the expected production of GFP and DPRs, respectively (Figure S1E). Notably, under these conditions, C9-HRE mRNA decayed rapidly, in contrast to the stable control GFP mRNA or the endogenous repeat-free *C9orf72* or *GAPDH* mRNAs present within the HEK293T cytosolic lysate (Figure 1I). Moreover, when the translation buffer was omitted, both synthetic mRNAs remained relatively stable, indicating that active translation is a prerequisite for the accelerated degradation of C9-HRE mRNA in vitro (Figure S1F). Together, our data demonstrate that RAN translation of the C9-HRE mRNA directly triggers its instability and translation-dependent decay.

### 2.2. RNA Exosome Spatially Associates with the Ribosome-C9-HRE mRNA Complex During RAN Translation

**Figure 2:**
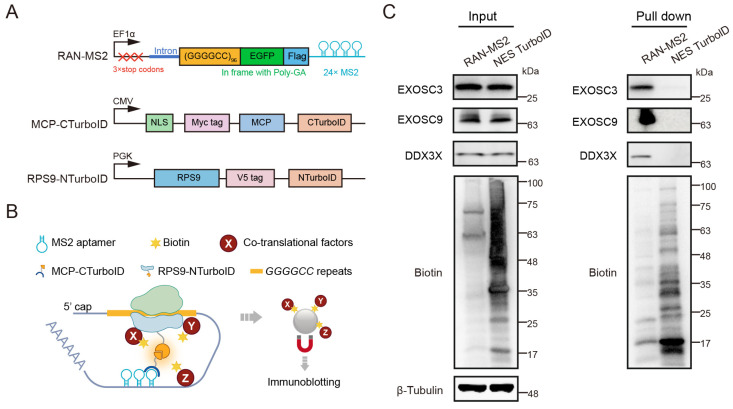
The RNA exosome associates with the translating ribosome-mRNA complex during RAN translation. (**A**) Scheme of the C9-HRE reporter (RAN-MS2), in which the *GGGGCC* repeats precede 24 × MS2 stem-loops, MCP-CTurboID, and RPS9-NTurboID. Detailed reporter sequences are provided in Table S1. (**B**) Schematic of the proximal labeling of co-translational regulators of C9-HRE mRNA during RAN translation. Interaction between MCP-CTurboID bound to the C-terminus of the C9-HRE mRNA and the elongating ribosome enabled reconstitution of functional TurboID. Biotinylated proteins were captured using streptavidin beads and analyzed by immunoblotting. (**C**) Biotinylated proteins in the inputs and pulldowns from ((**B**), RAN-MS2) were analyzed using immunoblotting with the indicated antibodies. Cells expressing cytosolic full-length TurboID (NES-TurboID) served as a non-specific control.

The RNA exosome has been reported to degrade C9-HRE mRNA in the nucleus and play a critical role in regulating the co-translational decay of mRNAs containing stalled ribosomes [5,44]. Therefore, we hypothesized that the RNA exosome may be responsible for the co-translational decay of C9-HRE mRNA during RAN translation. To determine whether the RNA exosome can recognize and engage translating C9-HRE mRNA, we employed a dimerization-dependent split-TurboID system [48], in which the promiscuous biotinylating enzyme TurboID was split into two inactive fragments. Specifically, the N-terminal fragment (NTurboID) was fused to the 40S ribosomal protein RPS9, while the C-terminal fragment (CTurboID) was fused to the MS2 capsid protein (MCP) (Figure 2A). Additionally, we modified the C9-HRE reporter by inserting MS2 stem-loops into the 3′ UTR sequence (Figure 2A and Table S1). In this system, MCP-CTurboID binds C9-HRE mRNA via high-affinity MCP-MS2 interactions (Figure 2B). During RAN translation, this complex is brought into proximity with elongating ribosomes, enabling dimerization of NTurboID-RPS9 and MCP-CTurboID. This reconstitution of TurboID activity results in the selective biotinylation of proteins associated with the translating ribosome-C9-HRE mRNA complex. To minimize non-specific labeling in the cytoplasm, the MCP fusion protein was tagged with a nuclear localization signal (NLS).

We confirmed that the addition of the MS2 sequence to the C9-HRE reporter did not impair RAN translation or DPR production in cells stably expressing the reporter (Figure S2A,B). Furthermore, we stably introduced both NTurboID-RPS9 and MCP-CTurboID into the reporter cell line via lentiviral transduction. We verified their expression and confirmed their predominantly cytoplasmic and nuclear distributions, respectively (Figure S2C,D). We also generated a cytoplasmically restricted, full-length TurboID by fusing it to a nuclear export signal (NES-TurboID), which served as a non-specific control. Following biotin supplementation, cytoplasmic fractions were isolated, and biotinylated proteins were enriched using streptavidin beads. As a positive control, DDX3X, a known RAN translation regulator [49,50], was specifically enriched in the split-TurboID group compared to the NES-TurboID control, confirming the specificity and reliability of the assay (Figure 2C). Given the critical function of the RNA exosome in co-translational mRNA decay, and because EXOSC3 and EXOSC9 represent critical structural components of the exosome cap and central channel, respectively, we examined their proximity labeling during active RAN translation. Indeed, immunoblot analysis of these targeted candidates revealed that both subunits were also specifically enriched in the split-TurboID group (Figure 2C). Together, these data support a model in which the RNA exosome is recruited to and spatially associates with the ribosome-C9-HRE mRNA complex during RAN translation in the cytoplasm.

### 2.3. RNA Exosome Mediates RAN Translation-Coupled mRNA Decay and Prevents DPR Production

**Figure 3:**
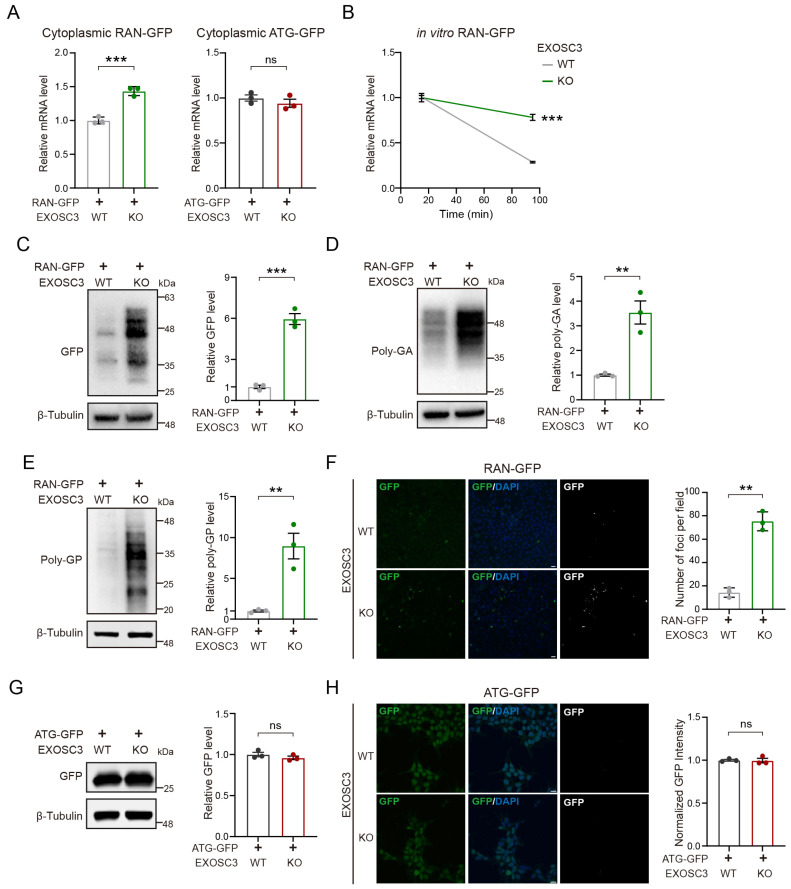
The RNA exosome mediates RAN translation-coupled mRNA decay. (**A**) Stable cell lines expressing RAN-GFP or ATG-GFP, with or without EXOSC3 knockout (KO), were subjected to subcellular fractionation. Cytoplasmic fractions were analyzed by RT-qPCR to quantify mRNA abundance of C9-HRE or ATG-GFP (n = 3). WT, wild-type. (**B**) Synthetic C9-HRE mRNA was incubated with soluble lysates obtained from either wild-type (WT) or EXOSC3 KO cells. Translation buffer was subsequently added for 15 or 90 min to initiate in vitro RAN translation, and C9-HRE mRNA levels were analyzed (n = 3). (**C**–**E**) Immunoblot analysis and quantification of DPR levels in RAN-GFP stable cell lines with or without EXOSC3 KO, using antibodies against GFP, poly-GA, or poly-GP (n = 3). (**F**) Representative confocal images of poly-GA-GFP in RAN-GFP stable cell lines with or without EXOSC3 KO. GFP signals are shown in both green and as a high-contrast binary image to enhance visibility. The number of GFP-positive inclusions per field in each group was quantified (n = 3 independent experiments, >5 fields analyzed per experiment). Scale bar: 10 μm. (**G**) Immunoblot analysis and quantification of GFP levels in ATG-GFP stable cell lines with or without EXOSC3 KO (n = 3). (**H**) Representative confocal images of GFP and quantification of GFP fluorescence intensities in ATG-GFP stable cells with or without EXOSC3 KO (n = 3 independent experiments, >5 fields analyzed per experiment). GFP signals are shown in both green and as a binary image. Scale bar: 10 μm. Data are presented as mean ± SEM. Statistical significance was determined using two-way ANOVA for (**B**) and unpaired two-sided Student’s *t*-test for (**A**,**C**–**H**); ** *p* < 0.01; *** *p* < 0.001; ns, not significant.

We next sought to investigate the functional role of the RNA exosome in regulating RAN translation-coupled C9-HRE mRNA decay. EXOSC3 is an essential component of the RNA exosome cap complex, required for RNA substrate recruitment and stabilization of the exosome [40]. Mutations in *EXOSC3* are associated with pontocerebellar hypoplasia and spinal motor neuron degeneration [51,52]. Therefore, using CRISPR-Cas9 technology, we knocked out EXOSC3 to disrupt RNA exosome function in stable cell lines expressing either the control GFP or C9-HRE reporter (Figure S3A). Loss of EXOSC3 led to an approximate two-fold increase in total C9-HRE mRNA levels, whereas control GFP mRNA levels remained unaffected (Figure S3B). Furthermore, subcellular fractionation revealed a specific 1.5-fold increase in cytoplasmic C9-HRE mRNA, the transcript directly responsible for RAN translation, with no corresponding change observed in the control GFP mRNA (Figure 3A).

To directly assess the role of the RNA exosome in RAN translation-coupled decay of C9-HRE mRNA, we performed an in vitro RAN translation assay using cytoplasmic fractions from control or EXOSC3 KO cells. Translation-coupled mRNA degradation was significantly attenuated in the EXOSC3 KO group, confirming the critical role of the RNA exosome in this process (Figure 3B).

We then evaluated the impact of EXOSC3 KO on DPR production. Western blot analysis using antibodies against GFP (in-frame with poly-GA), poly-GA, and poly-GP revealed a significant increase in DPR levels in EXOSC3 KO cells (Figure 3C–E). Consistently, fluorescence imaging showed a marked increase in the number of cytoplasmic GFP-positive inclusions in EXOSC3-deficient cells (Figure 3F). In contrast, conventional translation and GFP production in control GFP cells were unaffected by EXOSC3 loss, further supporting the specific role of EXOSC3 in regulating RAN translation (Figure 3G,H).

Interestingly, the 5- to 9-fold increase in DPR protein levels was disproportionately larger than the 1.5-fold increase in cytoplasmic *C9orf72* HRE mRNA (Figure 3A,C–E). To address this, we calculated normalized RAN translation efficiencies for poly-GA and poly-GP reading frames by dividing the fold-change in DPR production by the relative cytoplasmic *C9orf72* HRE mRNA levels (Figure S3C). This analysis revealed an elevated RAN translation efficiency in EXOSC3 KO cells, suggesting that the translation-coupled C9-HRE mRNA decay mediated by the RNA exosome is also critical to suppress RAN translation efficiency and prevent excessive DPR production.

### 2.4. EXOSC3 Promotes RAN Translation-Coupled C9-HRE mRNA Decay

**Figure 4:**
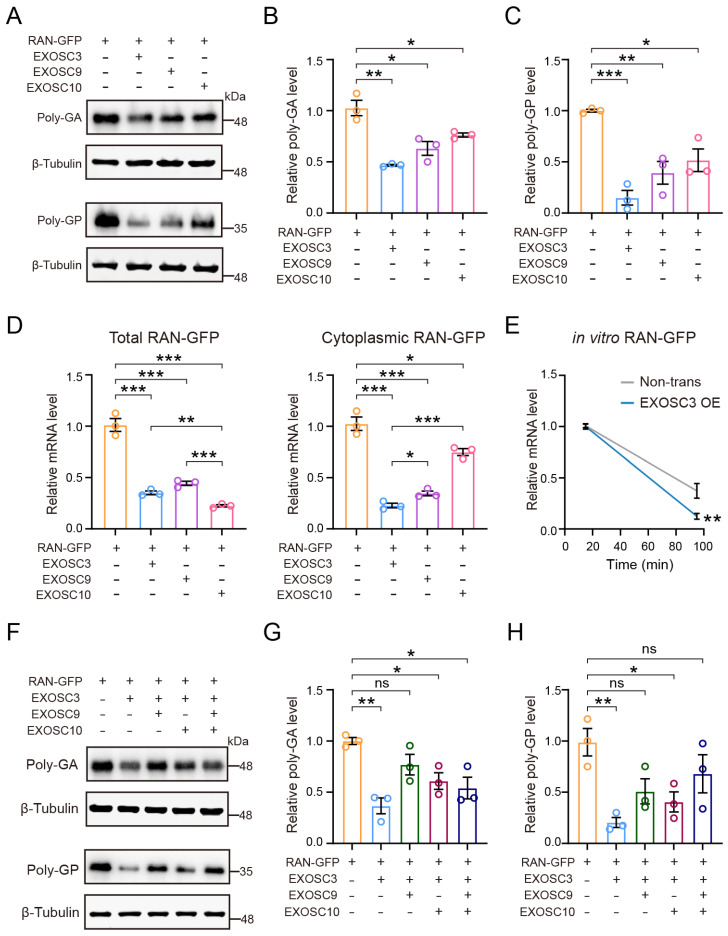
Overexpression of EXOSC3 specifically enhances RAN translation-coupled C9-HRE mRNA decay. (**A**–**C**) RAN-GFP stable cells were transfected with equal amounts of EXOSC3, EXOSC9, or EXOSC10 constructs for 48 h. DPR levels in these cells were analyzed and quantified using immunoblotting with antibodies against poly-GA and poly-GP (n = 3). (**D**) RAN-GFP stable cells transfected with the indicated constructs were subjected to subcellular fractionation. C9-HRE mRNA abundances in the whole cells (total) or the cytoplasmic fractions were analyzed by RT-qPCR (n = 3). (**E**) Synthetic C9-HRE mRNA was incubated with soluble lysates obtained from cells with or without EXOSC3 overexpression (OE). Translation buffer was subsequently added for 15 or 90 min to initiate in vitro RAN translation, and C9-HRE mRNA levels were analyzed (n = 3). Non-trans: non-transfection. (**F**–**H**) RAN-GFP stable cells were transfected with EXOSC3 alone or in combination with EXOSC9 and/or EXOSC10 for 48 h. Each construct was transfected at an equal amount, and the total plasmid amount of each group was equalized using an empty vector. DPR levels were analyzed and quantified by immunoblotting using antibodies against poly-GA and poly-GP (n = 3). Data are mean ± SEM. Statistical significance was determined using two-way ANOVA for (**E**) and unpaired two-sided Student’s *t*-test for (**B**–**D**,**G**,**H**); * *p* < 0.05; ** *p* < 0.01; *** *p* < 0.001; ns, not significant.

During RNA degradation, the 3′ end of a single-stranded RNA substrate is recruited by the cap subunits and threaded through the central channel. We next examined whether enhancing RNA exosome activity suppresses RAN translation efficiency and DPR production. To test this, we overexpressed EXOSC3, an RNA-binding domain-containing subunit of the exosome cap, and EXOSC9, a subunit of the central channel (Figure S4). Additionally, we overexpressed EXOSC10, a nuclear catalytic subunit of the exosome previously implicated in C9-HRE mRNA degradation [44], as a control (Figure S4). Overexpression of all three exosome components significantly reduced DPR production, with EXOSC3 exhibiting the strongest inhibition (Figure 4A–C). Analysis of C9-HRE mRNA levels revealed that EXOSC10 overexpression maximally reduced total C9-HRE mRNA levels, consistent with its role in nuclear decay (Figure 4D). Conversely, EXOSC3 overexpression caused the greatest decrease in cytoplasmic C9-HRE mRNA levels, aligning with its potent suppression of DPR production (Figure 4D). Critically, we directly confirmed that EXOSC3 overexpression accelerated the RAN translation-coupled decay of C9-HRE mRNA, demonstrating its dominant role in inhibiting RAN translation.

We next tested whether co-overexpression of EXOSC3 with EXOSC9 and/or EXOSC10 would produce a synergistic inhibitory effect. Although EXOSC3 overexpression alone was sufficient to suppress RAN translation, co-expression with EXOSC9, EXOSC10, or both did not further enhance this effect (Figure 4F–H). These findings suggest that EXOSC3 is a key limiting factor in RAN translation-associated *C9orf72* HRE mRNA decay and DPR suppression.

### 2.5. EXOSC3 Is Critical for Suppressing RAN Translation in iPSC-Derived Neurons

**Figure 5:**
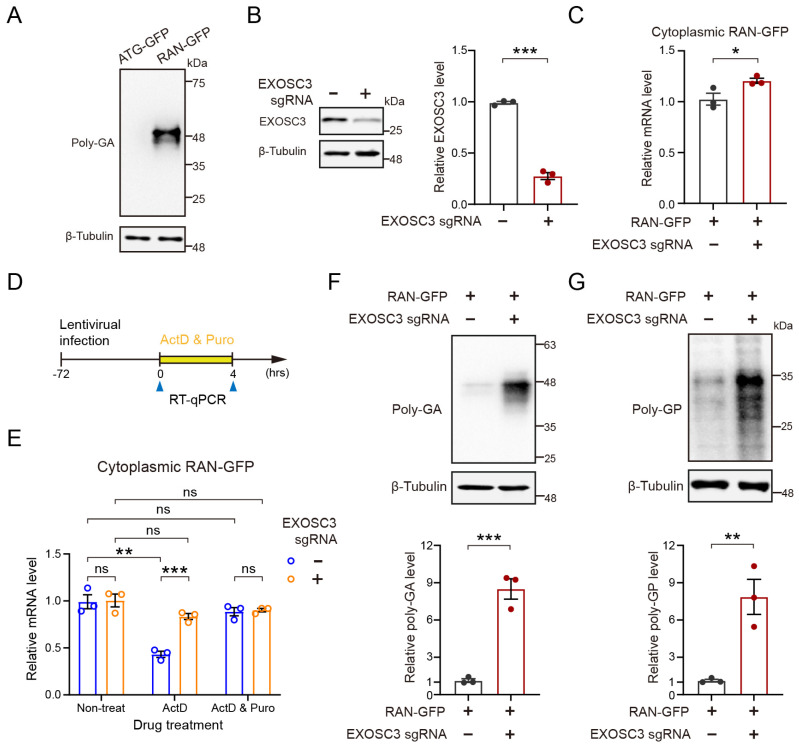
EXOSC3 is critical for suppressing RAN translation in i3Neurons. (**A**) RAN translation of the RAN-GFP reporter in i3Neurons. i3Neurons were transduced with a RAN-GFP lentivirus for 72 h prior to harvesting. Poly-GA levels were analyzed by immunoblotting. (**B**) Validation of EXOSC3 knockdown in i3Neurons. i3Neurons were transduced with a lentivirus encoding Cas9 and an sgRNA targeting EXOSC3 for 72 h prior to harvesting. EXOSC3 protein levels were analyzed and quantified using immunoblotting (n = 3). (**C**) i3Neurons expressing the RAN-GFP reporter, with or without EXOSC3 knockdown, were subjected to subcellular fractionation. C9-HRE mRNA abundances in the cytoplasmic fractions were analyzed using RT-qPCR (n = 3). (**D**,**E**) Treatment scheme: i3Neurons were transduced with RAN-GFP via lentiviral infection for 72 h, followed by a 4 h treatment of ActD in the presence or absence of puromycin. C9-HRE mRNA abundances in the cytoplasmic fractions were analyzed using RT-qPCR (n = 3). (**F**,**G**) Immunoblot analysis and quantification of DPR levels in i3Neurons expressing the RAN-GFP reporter, with or without EXOSC3 knockdown, using antibodies against poly-GA or poly-GP (n = 3). Data are presented as mean ± SEM. Statistical significance was determined using unpaired two-sided Student’s *t*-test; * *p* < 0.05; ** *p* < 0.01; *** *p* < 0.001; ns, not significant.

Given that RAN translation of C9-HRE contributes to neuronal toxicity in C9-ALS/FTD, we next investigated the role of EXOSC3 in human iPSC-derived neurons. To this end, we generated an iPSC line harboring a doxycycline-inducible NGN2 cassette inserted into the *AAVS1* locus via CRISPR/Cas9-mediated homologous recombination (Figure S5A) [53,54]. These engineered iPSCs (referred to as i3N iPSCs) can be rapidly differentiated into functional excitatory neurons (i3Neurons), a widely used model for studying neurodegenerative disease mechanisms, including RAN translation [54,55,56]. We confirmed efficient differentiation of i3N iPSCs into i3Neurons (Figure S5B). We then introduced the C9-HRE reporter into the i3Neurons via lentiviral transduction. The expression of poly-GA confirmed active RAN translation in these cells (Figure 5A).

To assess the role of EXOSC3, we attenuated its expression via lentiviral CRISPR/Cas9 targeting *EXOSC3* (Figure 5B). This reduction in EXOSC3 levels led to a moderate but significant increase in cytoplasmic C9-HRE mRNA levels (Figure 5C). To further investigate mRNA stability, cells were treated with ActD in the presence or absence of puromycin. Reduced EXOSC3 expression markedly impaired the decay of C9-HRE mRNA (Figure 5D,E). Notably, puromycin treatment eliminated the difference in decay rates between wild-type and EXOSC3-knockdown cells, demonstrating that this EXOSC3-mediated clearance is translation-dependent (Figure 5D,E). Finally, we measured DPR levels in i3Neurons with or without EXOSC3 knockdown and found that loss of EXOSC3 significantly increased DPR production (Figure 5F,G). Moreover, the disproportionately large increase in DPR levels, relative to the modest rise in cytoplasmic C9-HRE mRNA, suggests that RAN translation efficiency is enhanced upon EXOSC3 depletion (Figure S5C). Together, these findings highlight the essential role of EXOSC3 in regulating RAN translation-coupled mRNA decay and preventing pathological DPR accumulation in neurons.

## 3. Discussion

The pathogenesis of C9-ALS/FTD is currently attributed to three primary mechanisms: (1) C9orf72 haploinsufficiency, wherein the repeat expansion inhibits transcription, thereby impairing C9orf72-dependent functions including autophagolysosomal flux, inflammatory responses, and energy metabolism; (2) RNA toxicity, driven by the formation of intranuclear C9-HRE RNA foci that sequester essential ribonucleoproteins; and (3) the accumulation of DPRs generated via RAN translation [17,57]. Among these interconnected mechanisms, RAN translation has emerged as a key driver of disease onset and progression. DPRs generated from C9-HRE transcripts exert broad and potent neurotoxicity by impairing RNA biogenesis, inducing ER stress, disrupting liquid–liquid phase separation (LLPS) and membraneless organelle dynamics, compromising mitochondrial function, and blocking both nucleocytoplasmic transport and the proteasome [24,25,58,59,60,61]. The extensive structural and functional damage induced by these RAN translation products underlies their central role in motor and cortical neuron degeneration. Consistent with this view, recent preclinical studies have shown that suppression of RAN translation, without altering the C9-HRE sequence or its expression, can fully rescue pathological and behavioral phenotypes in C9-ALS/FTD mouse models [30]. In contrast, a clinical study using antisense oligonucleotides (ASOs) to reduce C9-HRE transcript levels failed to decrease DPR abundance and produced only limited efficacy against disease-associated pathology [62]. Thus, elucidating endogenous intracellular mechanisms that constrain this aberrant translation is of critical importance for therapeutic development. In this study, we discovered a quality control (QC) pathway that surveils C9-HRE mRNA during RAN translation and limits DPR production.

To maintain cellular proteostasis, eukaryotic cells have evolved a sophisticated network of co-translational quality control mechanisms, including NMD, NSD, and NGD, which act synergistically to resolve translational errors and eliminate aberrant mRNAs and nascent peptides [5,6]. NGD is triggered by elongating ribosomes stalled at stable RNA secondary structures, NSD targets transcripts lacking canonical stop codons, and NMD degrades mRNAs containing premature termination codons. In the context of C9-ALS/FTD pathogenesis, extensive *GGGGCC* repeat arrays form highly stable and complex secondary structures (e.g., G-quadruplexes and hairpins) that impede translating ribosomes [32,33]. Recent studies confirm that these QC pathways and their associated factors play critical roles in clearing the DPR products of RAN translation [34,35,36,37]. Manipulation of these QC factors directly influences the steady-state levels of DPRs and alters the severity of cellular toxicity, validating their protective and regulatory functions. However, while peptide-level QC mechanisms in RAN translation are relatively well characterized, mRNA-level surveillance during RAN translation-coupled QC remains elusive. Here, we demonstrated that the cytoplasmic RNA exosome directly engages the translating ribosome-C9-HRE mRNA complex to mediate RAN translation-coupled mRNA decay. These findings reveal a previously unrecognized layer of mRNA-level, co-translational regulation of DPR production, thereby expanding the current paradigm of co-translational QC.

The RNA exosome is central to the clearance of defective transcripts targeted by co-translational QC pathways, mediating 3′-to-5′ RNA degradation [38,39]. Structurally, the RNA exosome comprises an unstable ring structure formed by six subunits (EXOSC4–EXOSC9), capped by a trimeric complex of RNA-binding subunits (EXOSC1, EXOSC2, and EXOSC3), which stabilizes the complex and facilitates cofactor recruitment and substrate recognition [40]. Mutations in these cap subunits are associated with neurodegenerative phenotypes [63,64]. For example, mutations in EXOSC2 cause a complex neurological disease known as Short stature, Hearing loss, Retinitis pigmentosa and distinctive Facies (SHRF Syndrome, OMIM #617763) [65]. Mutations in EXOSC3 are linked to pontocerebellar hypoplasia type 1b (PCH1b), an autosomal-recessive neurodegenerative disease characterized by significant atrophy of the pons and cerebellum, Purkinje cell abnormalities, and degeneration of spinal motor neurons (OMIM #614687) [51,52]. Notably, the vast majority of known pathogenic mutations across all RNA exosome subunits localize to EXOSC3, underscoring its indispensable role in neuronal RNA surveillance and survival [63]. In this study, we further demonstrate the critical role of EXOSC3 in engaging RAN translation in both cellular models and iPSC-derived neurons, where it facilitates the rapid degradation of pathogenic *GGGGCC* mRNA. Importantly, the overexpression of EXOSC3 alone is sufficient to reduce cytoplasmic C9-HRE mRNA levels, suppress RAN translation, and decrease DPR production. Collectively, our findings identify a critical endogenous, co-translational neuroprotective mechanism operating at the mRNA level and highlight exosome-mediated RNA decay as a promising therapeutic target for C9-ALS/FTD. While our data robustly demonstrate that EXOSC3 mediates translation-dependent decay of C9-HRE mRNA, a limitation of the current study is distinguishing whether EXOSC3 strictly reduces RNA substrate availability or concurrently suppresses RAN translation efficiency through direct ribosomal interactions. Future biochemical studies utilizing polysome profiling or advanced single-molecule imaging techniques will be essential to dissect these mechanisms.

## 4. Materials and Methods

### 4.1. Plasmids

A RAN-GFP-MS2 reporter was constructed by inserting three stop codons upstream of a *C9orf72* intronic sequence containing 96 *GGGGCC* repeats to prevent canonical translation. An EGFP lacking the ATG start codon was subcloned downstream of the *GGGGCC* repeats in frame with poly-GA, followed by 24 copies of MS2 stem-loop sequences, and cloned in the Lenti-EF-vector for constitutive expression. MCP and RPS9 were cloned into two different Lenti vectors using the Gateway recombination cloning system (Thermo Fisher, Waltham, MA, USA). The TurboID-NES reporter was constructed by cloning into a lentiviral expression vector. LentiCRISPR v2 was obtained from Addgene (#49535, Watertown, MA, USA), and annealed oligonucleotides encoding a sgRNA targeting *EXOSC3* was ligated into the BsmBI-digested vector. RAN-GFP and ATG-GFP reporters were constructed under the EF1α promoter. The RAN-GFP construct contains a *C9orf72* intronic sequence with *GGGGCC* repeats followed by an ATG-deficient EGFP in the poly-GA frame, whereas the ATG-GFP construct contains a Kozak sequence and a canonical ATG to drive EGFP expression. The RAN-Flag reporter was further constructed by replacing EGFP with a Flag tag and the EF1α promoter with a T7 promoter. The cDNAs encoding EXOSC3, EXOSC9, and EXOSC10 were synthesized and subsequently recombined into pLenti-CMV-Puro-DEST (w118-1) vector using the Gateway cloning system [66].

### 4.2. iPSC Culture and i3Neuron Differentiation

A healthy iPSC line was maintained on Matrigel (BD)-coated 6-well plates in mTeSR Plus medium (STEMCELL Technologies, Vancouver, BC, Canada) using a feeder-free culture protocol [67]. The i3N iPSC line was generated via CRISPR/Cas9-mediated homologous recombination, adapted from previous protocols with minor modifications [53,54]. Briefly, 1 × 10^6^ iPSCs were resuspended in the P3 Primary Cell 4D-Nucleofector X Kit L (Lonza, Walkersville, MD, USA) and nucleofected using a 4D-Nucleofector system (Lonza, Walkersville, MD, USA). Cells were co-nucleofected with a donor plasmid encoding TRE-NGN2 (pUCM-AAVS1-TO-hNGN2, Addgene #105840; Watertown, MA, USA) and a CRISPR/Cas9 plasmid expressing an AAVS1-targeting sgRNA (pLentiCRISPR-Puro-sgAAVS1, #187818, Addgene, Watertown, MA, USA). Three days post-nucleofection, cells were subjected to 1 μg/mL puromycin selection for 72 h. To excise the selection marker, cells were subsequently transfected with pCAG-Cre-IRES2-GFP (#26646, Addgene, Watertown, MA, USA). Single colonies were manually picked, expanded, and validated by PCR-based genotyping.

Differentiation of i3N iPSCs into i3Neurons was performed using a rapid, doxycycline-inducible NGN2-based protocol. At day −3, i3N iPSCs were dissociated and seeded onto Geltrex-coated plates in DMEM/F12 medium supplemented with N2, NEAA, BDNF, and NT3. Doxycycline (2 μg/mL, Selleck, Houston, TX, USA) was added to induce NGN2 expression. A ROCK inhibitor (Y-27632) (10 μM, Selleck, Houston, TX, USA) was included during the first 24 h to enhance cell survival. At day 0, the induced cells were dissociated into single cells using Accutase (STEMCELL Technologies, Vancouver, BC, Canada) and re-plated at the desired density onto poly-D-lysine- and laminin-coated plates. Cells were maintained in a neuronal maturation medium comprising Neurobasal medium supplemented with B27, BDNF, NT3, and laminin. Half-medium changes were performed every 2–3 days to support neuronal survival, neurite outgrowth, and functional maturation.

### 4.3. Cell Culture and Treatments

HEK293T cells were cultured in Dulbecco’s Modified Eagle Medium (DMEM) supplemented with 10% (*v*/*v*) fetal bovine serum (FBS), 100 U/mL penicillin, and 100 μg/mL streptomycin. For transient expression, cells were transfected with ATG-GFP or RAN-GFP plasmid DNA using the Lipo8000 transfection reagent (Beyotime, Shanghai, China) according to the manufacturer’s instructions, and incubated for 12 h post-transfection. To prepare lentiviruses, HEK293T cells at ~80% confluency were co-transfected with a lentiviral transfer vector and the packaging plasmids psPAX2, and pMD2.G at a 3:2:1 ratio using Lipofectamine 3000 (Thermo Fisher, Waltham, MA, USA). After 48 h, viral supernatants were harvested, filtered through a 0.45-μm membrane, concentrated via overnight precipitation at 4 °C, and pelleted by centrifugation at 1600× *g* for 1 h.

To establish stable cell lines, HEK293T cells or iPSCs were transduced with lentiviruses for 72 h. Subsequently, the cells were selected using 1 μg/mL puromycin for an additional 72 h. Single colonies were then picked and expanded. Cells were maintained in complete medium without puromycin for at least 10 passages prior to further analysis to minimize potential effects of puromycin on translation. For drug treatments, cells were exposed to 20 μg/mL actinomycin D (ActD) alone or in combination with 1 μg/mL puromycin for 4 or 8 h. Following treatment, cells were harvested, and total RNA was extracted using TRIzol reagent (Thermo Fisher, Waltham, MA, USA) for subsequent real-time RT-qPCR analysis.

### 4.4. Subcellular Fractionation

Cells cultured in 6-well plates were subjected to the indicated experimental conditions. Cells were washed once with 1 mL of ice-cold PBS and harvested by scraping into 300 μL of ice-cold cytoplasmic lysis buffer (CLB; 25 mM HEPES, pH 7.9, 5 mM KCl, 0.5 mM MgCl_2_, and 0.5% NP-40), freshly supplemented with EDTA-free protease inhibitor cocktail (Roche, Burlington, MA, USA) and 200 U/mL RNaseOUT (Thermo Fisher, Waltham, MA, USA). Lysates were transferred to RNase-free tubes, incubated on ice for 5 min to ensure complete lysis, and centrifuged at 500× *g* for 5 min at 4 °C. The supernatants were collected as the cytoplasmic fractions, while the pellets were collected as the nuclear fractions.

### 4.5. In Vitro Transcription

Plasmids were extracted and linearized using the restriction enzyme XhoI (NEB, Ipswich, MA, USA) to serve as templates for in vitro transcription. Linearized DNA was transcribed using the HiScribe T7 High Yield RNA Synthesis Kit (NEB, Ipswich, MA, USA), with 3′-O-Me-m7GpppG anti-reverse cap analog (ARCA) added at four times the concentration of GTP. The 20 μL T7 reactions containing 1 μg DNA templates were incubated at 37 °C in a PCR machine for 2 h. Following transcription, the DNA template was degraded by incubation with DNase I at 37 °C for 15 min. mRNAs were cleaned and concentrated with RNA Clean and Concentrator Kit (Zymo, Irvine, CA, USA). The concentration and purity of the mRNA were determined using a NanoDrop spectrophotometer, and the size and quality of synthesized mRNAs were verified on a denaturing formaldehyde RNA gel.

### 4.6. In Vitro Transcribed RNA Degradation Assays

HEK293T cells were seeded in 6-well plates. Following overnight incubation, DMEM was exchanged, and 500 ng/mL of each in vitro transcribed RAN-Flag or ATG-Flag RNA was transfected into the cells with Opti-MEM and lipofectamine 3000 (Thermo Fisher, Waltham, MA, USA) for 12 h. Cells were then cultured for 8 h in fresh complete medium with or without puromycin (final concentration, 1 μg/mL). Cells were then washed with PBS and subjected to RT-qPCR analysis.

### 4.7. Cell Lysates Preparation and In Vitro Translation

The method used for in vitro translation analysis was based on a previous report with minor modifications [47]. Briefly, HEK293T cells were harvested by trypsinization and centrifugation. The cell pellets were washed by resuspension in 5 packed cell volumes (PCV) of PBS and centrifuged at 1800× *g* for 10 min at 4 °C. Subsequently, cells were rapidly resuspended in 5 PCV of hypotonic buffer and centrifuged at 1800× *g* for 5 min at 4 °C. The PCV was measured, and cells were swollen on ice for 30 min in 3 PCV of RNase-free hypotonic buffer containing 10 mM HEPES-KOH (pH 7.6), 10 mM potassium acetate, 0.5 mM magnesium acetate, 5 mM DTT, and EDTA-free protease inhibitor cocktail. Cell disruption was achieved by 10 slow strokes using a glass homogenizer with a loose pestle on ice. The homogenate was incubated on ice for an additional 20 min and clarified by centrifugation at 10,000× *g* for 10 min at 4 °C. The resulting supernatant was flash-frozen in liquid nitrogen and stored in aliquots at −80 °C.

In vitro translation reactions were assembled using 75% (*v*/*v*) cytoplasmic lysate supplemented to final concentrations of 20 mM HEPES-KOH (pH 7.6), 44 mM potassium acetate, 2.2 mM magnesium acetate, 2 mM DTT, 20 mM creatine phosphate (Roche, Burlington, MA, USA), 0.1 μg/μL creatine kinase, 0.1 mM spermidine, 0.1 mM of each amino acid, and 0.1 U/μL RNaseOUT. Reactions were initiated by adding 5 nM purified, in vitro transcribed reporter mRNA that had been denatured at 65 °C for 5 min and immediately chilled on ice to minimize non-specific secondary structure formation. Reactions were incubated at 33 °C for 15 or 90 min and terminated by placing the tubes on ice.

### 4.8. RNA Extraction and Real-Time RT-qPCR

Total RNA was extracted from cell pellets using TRIzol reagent or from cytoplasmic fractions using TRIzol LS reagent, followed by chloroform phase separation and isopropanol precipitation in the presence of GlycoBlue. RNA pellets were washed twice with 75% ethanol, dissolved in RNase-free water, and quantified using a NanoDrop spectrophotometer (Thermo Scientific, Waltham, MA, USA). First-strand cDNA was synthesized using the Evo M-MLV cDNA Synthesis Kit (Accurate Biology, Changsha, China). Quantitative PCR was performed using SYBR Green Pro Taq HS Premix (Accurate Biology, Changsha, China) on a QuantStudio 5 Real-Time PCR System (Thermo Fisher, Waltham, MA, USA). TUBB or 18S rRNA was used as the internal control. Relative RNA levels were calculated using the ΔΔCt method. Primer sequences are listed in Table S2.

### 4.9. Split-TurboID Proximity Labeling Assay

For proximity labeling, stable cell lines cultured in 15 cm dishes were supplemented with biotin at a final concentration of 50 μM and incubated for 6 h. Cells were then washed once with ice-cold PBS and lysed in 1 mL cytoplasmic lysis buffer supplemented with 1× protease inhibitor cocktail and PMSF. Lysates were collected by scraping, followed by centrifugation at 500× *g* for 10 min at 4 °C. The resulting supernatant was mixed with five volumes of pre-chilled acetone and incubated at −30 °C overnight to precipitate proteins. The next day, samples were centrifuged at 3500× *g* for 5 min at 4 °C, and the protein pellets were washed twice with pre-chilled methanol and air-dried for 10 min. Pellets were resuspended in 1 mL RIPA buffer (50 mM Tris pH 8.0, 150 mM NaCl, 0.1% SDS, 1% NP40, and 1% sodium deoxycholate) supplemented with 1× protease inhibitor cocktail. Pre-equilibrated streptavidin magnetic beads were added to the lysates and incubated overnight at 4 °C with rotation to capture biotinylated proteins. The following day, beads were collected using a magnetic rack and the supernatant was removed. To minimize nonspecific binding, beads were washed sequentially with ice-cold RIPA buffer, 6 M urea, and PBS. After washing, beads were resuspended in RIPA buffer and boiled at 95 °C to elute bound proteins. Input and bead fractions were subsequently analyzed by SDS-PAGE and immunoblotting to assess the enrichment of biotinylated proteins.

### 4.10. Western Blot Analysis

Cells were lysed with RIPA Buffer supplemented with 6 M urea and 1× protease inhibitor cocktail. Cell lysates were mixed with SDS loading buffer 5× SDS sample buffer (250 mM Tris, pH 6.8, 750 mM NaCl, 5% NP40, 5% sodium deoxycholate, 10% SDS, 5% 2-mercaptoethanol, and 60 mM EDTA) and denatured at 98 °C for 10 min. Proteins were resolved by 10% SDS-PAGE and transferred onto nitrocellulose membranes (Cytiva, Marlborough, MA, USA). Membranes were blocked with 5% non-fat milk in Tris-buffered saline containing 0.1% Tween-20 (TBST) for 1 h at room temperature and subsequently probed with primary antibodies overnight at 4 °C. After washing with TBST, membranes were incubated with secondary antibodies (LI-COR Biosciences, Lincoln, NE, USA) and incubated for 2 h at room temperature. Immunoreactive bands were visualized with a dual-mode infrared imaging system (LI-COR Biosciences, Lincoln, NE, USA). Densitometric analysis was performed using FIJI software (version 1.54p), with target protein levels normalized to the corresponding loading controls. Primary antibodies for immunoblotting were as follows: GFP (AE078, ABclonal, Wuhan, China), poly-GA antibody (24492-1-AP, Proteintech, Wuhan, China), poly-GP (24494-1-AP, Proteintech, Wuhan, China), EXOSC3 (15062-1-AP, Proteintech, Wuhan, China), EXOSC9 (24470-1-AP, Proteintech, Wuhan, China), EXOSC10 (11178-1-AP, Proteintech, Wuhan, China), Flag tag (66008-4-Ig, Proteintech, Wuhan, China), Myc tag (2278S, Cell Signaling Technology, Danvers, MA, USA), V5 tag (AE017, ABclonal, Wuhan, China), β-Tubulin (TA503129, OriGene, Rockville, MD, USA).

### 4.11. Immunofluorescence

Cells grown on glass coverslips were fixed with 4% paraformaldehyde in PBS for 30 min at room temperature and permeabilized with 0.25% Triton X-100 in PBS for 10 min. After washing with PBS, cells were blocked with 1% BSA in PBST for 30 min at room temperature and then incubated with primary antibodies diluted in 1% BSA in PBST overnight at 4 °C. The following day, cells were washed three times with PBST for 5 min each and incubated with the appropriate secondary antibodies for 1 h at room temperature. After three washes with PBST and one wash with PBS, nuclei were counterstained with DAPI (0.1 mg/mL) for 15 min at room temperature, followed by three washes with 0.02% Tween-20 in PBS. Coverslips were mounted on glass slides using ProLong Diamond Antifade Mountant (Thermo Fisher, Waltham, MA, USA), and fluorescence images were acquired using an FV3000 confocal laser scanning microscope (Olympus, Tokyo, Japan). Primary antibodies used for the immunofluorescent staining were as follows: Flag tag (66008-4-Ig, Proteintech, Wuhan, China), Myc tag (2278S, Cell Signaling Technology, Danvers, MA, USA), V5 tag (AE017, ABclonal, Wuhan, China), TUJ1 (66375-1-Ig, Proteintech, Wuhan, China).

## Data Availability

The original contributions presented in this study are included in the article/Supplementary Material. Further inquiries can be directed to the corresponding authors.

## Supplementary Materials

The following supporting information can be downloaded at: https://www.mdpi.com/article/10.3390/ijms27156986/s1.
