# Supplementary material for: RAN Translation-Coupled Decay of the C9orf72 GGGGCC Repeat Transcript by the RNA Exosome Suppresses Dipeptide Repeat Production
Source: Int J Mol Sci. 2026 Aug 4;27(15):6986. doi: 10.3390/ijms27156986 (PMC13466912; doi:10.3390/ijms27156986)
Supplement: Supplementary file 1 [file ijms-27-06986-s001.zip › Supplementary Figures.pdf]

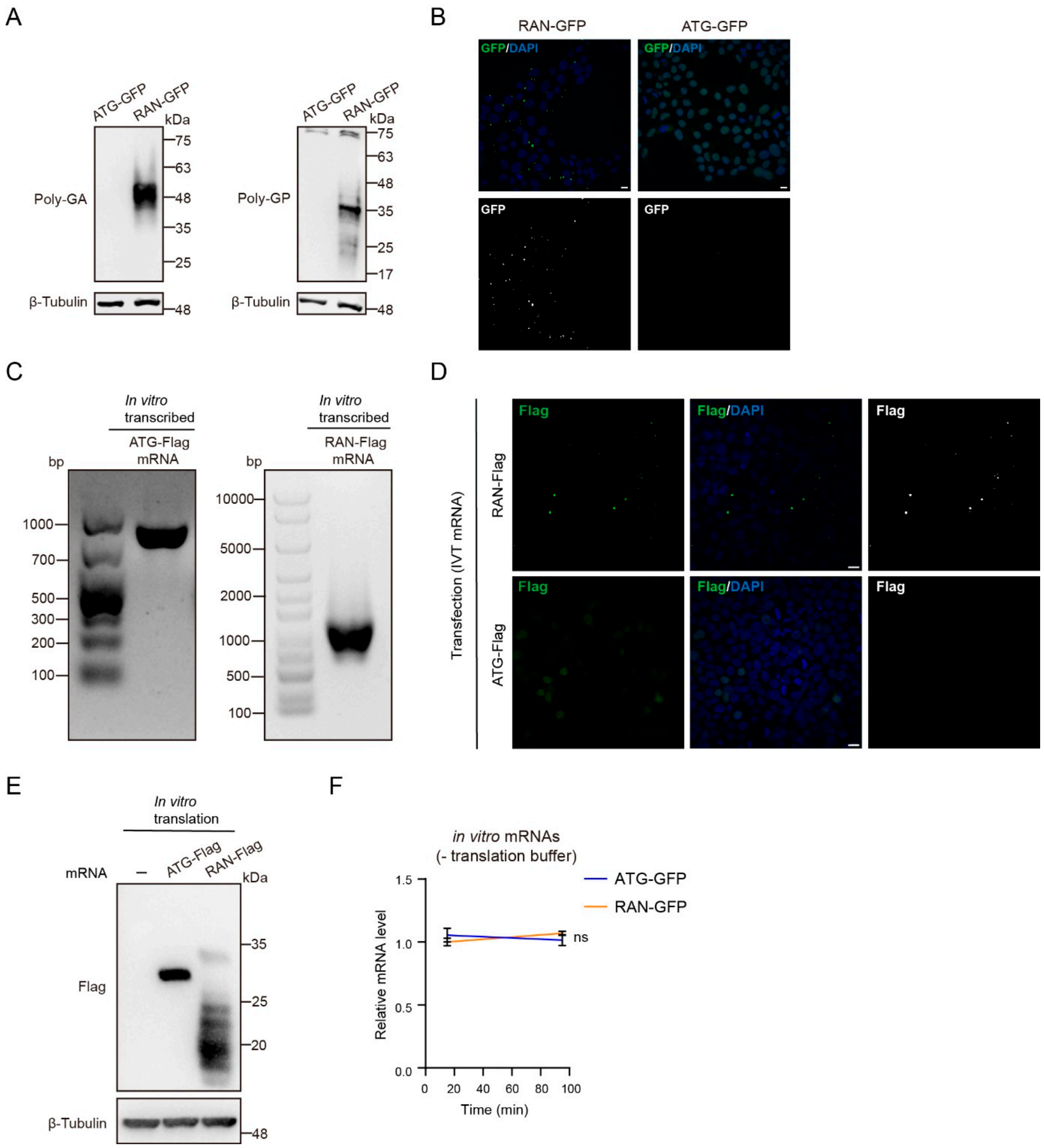

Figure S1. RAN translation of the RAN-GFP reporter in both cellular and *in vitro* translation systems.

A Immunoblot analysis of DPRs using antibodies against poly-GA or poly-GP in cells transfected with the RAN-GFP reporter for 48 hours.

B Representative confocal images showing poly-GA-GFP produced via RAN translation of the *GGGGCC* repeats. GFP signals are shown in both green and as a high-contrast binary image to enhance visibility. Poly-GA-GFP formed cytoplasmic inclusions, in contrast to the diffuse

GFP signal observed in the ATG-GFP control. Nuclei were visualized by DAPI (blue). Scale bar: 10 μm.

C Quality assessment of the *in vitro*-transcribed C9-HRE (RAN-Flag) and ATG-GFP mRNAs by gel electrophoresis, confirming expected transcript size and integrity.

D Validation of RAN translation of *in vitro*-transcribed C9-HRE mRNA in cells following transfection. Representative immunofluorescence confocal images demonstrate cytoplasmic inclusions of poly-GA-Flag in the RAN-GFP group. GFP signals are shown in both green and as a binary image. Nuclei were visualized by DAPI (blue). Scale bar: 10 μm.

E Validation of RAN translation in the *in vitro* translation assay. Immunoblot analysis of poly-GA-Flag and GFP-Flag, translated from synthetic RAN-Flag and ATG-Flag mRNAs, respectively, using an anti-Flag antibody.

F Reporter mRNA abundances in the *in vitro* translation mixtures for 15 and 90 minutes in the absence of translation buffer (n = 3).

Data are presented as mean ± SEM. Statistical significance was determined using two-way ANOVA; ns, not significant.

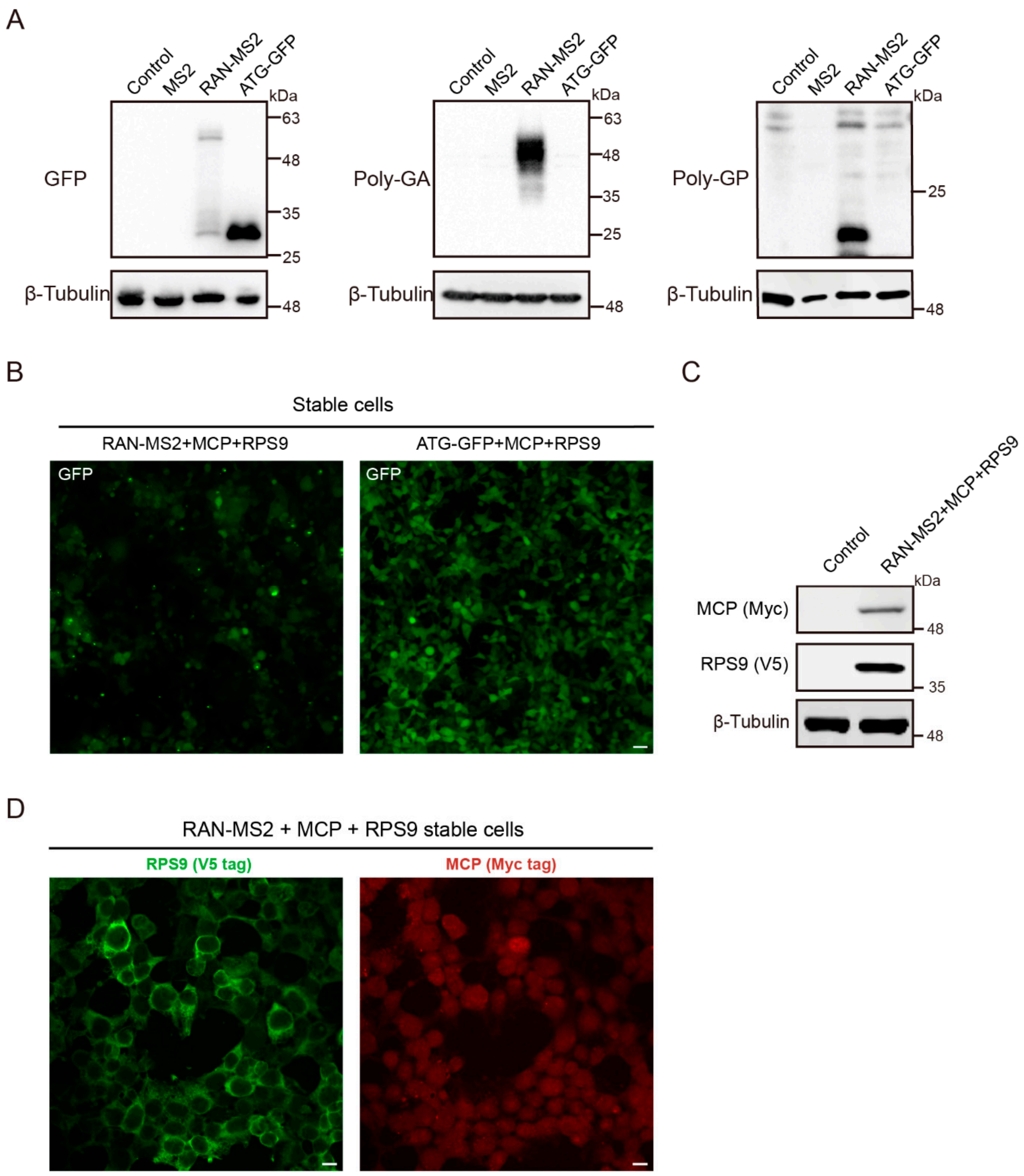

Figure S2. Validation of RAN translation and expression of split-TurboID proteins.

A Immunoblot analysis of DPRs using antibodies against GFP, poly-GA, or poly-GP in cells stably expressing the RAN-MS2 reporter. ATG-GFP cells served as a canonical translation control. Cells expressing an MS2-repeat-only reporter (MS2) served as a specificity control.

B Representative fluorescence images of cells stably co-expressing the RAN-MS2 reporter, MCP-CTurboID, and RPS9-NTurboID (RAN-MS2+MCP+RPS9), displaying robust cytoplasmic poly-GA-GFP inclusions. Cells stably co-expressing the canonical translation

reporter ATG-GFP, MCP-CTurboID, and RPS-NTurboID (ATG-GFP+MCP+RPS9) served as a control. Scale bar: 30 μm.

C, D Validation of the expression and subcellular localization of MCP-CTurboID (Myc tag) and RPS9-NTurboID (V5 tag) in the RAN-MS2+MCP+RPS9 stable cell line using immunoblotting and immunofluorescence. Scale bar: 10 μm.

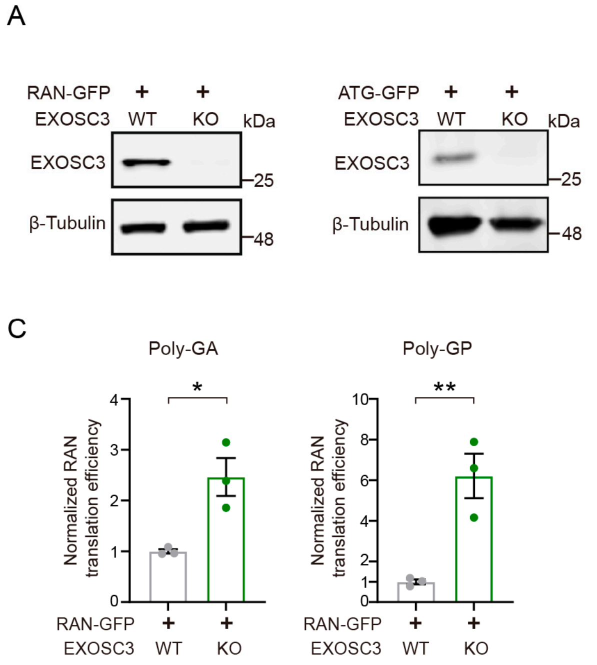

Figure S3. Validation of EXOSC3 knockout and its effects on total C9-HRE mRNA levels and RAN translation rates.

A Immunoblot analysis confirming the knockout of EXOSC3 in cells stably expressing RAN-GFP or ATG-GFP.

B Quantification of total mRNA abundances of ATG-GFP mRNA in ATG-GFP-expressing stable cells or C9-HRE mRNA in RAN-GFP-expressing stable cells, with or without EXOSC3 KO.

C Calculated RAN translation efficiencies for the production of poly-GA or poly-GP in RAN-GFP stable cells with or without EXOSC3 KO ($n = 3$).

Data are presented as mean ± SEM. Statistical significance was determined using unpaired two-sided Student's t-test; *$P < 0.05$; **$P < 0.01$; ***$P < 0.001$; ns, not significant.

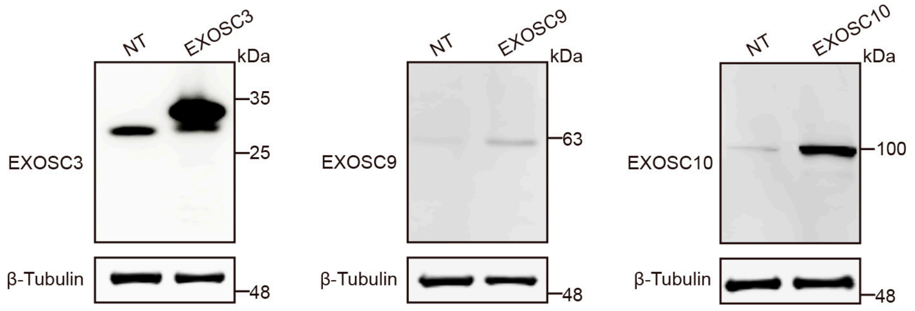

Figure S4. Validation of EXOSC3, EXOSC9, and EXOSC10 overexpression. HEK293 cells were transfected with the indicated constructs for 48 hours, followed by immunoblot analysis using antibodies against EXOSC3, EXOSC9, or EXOSC10.

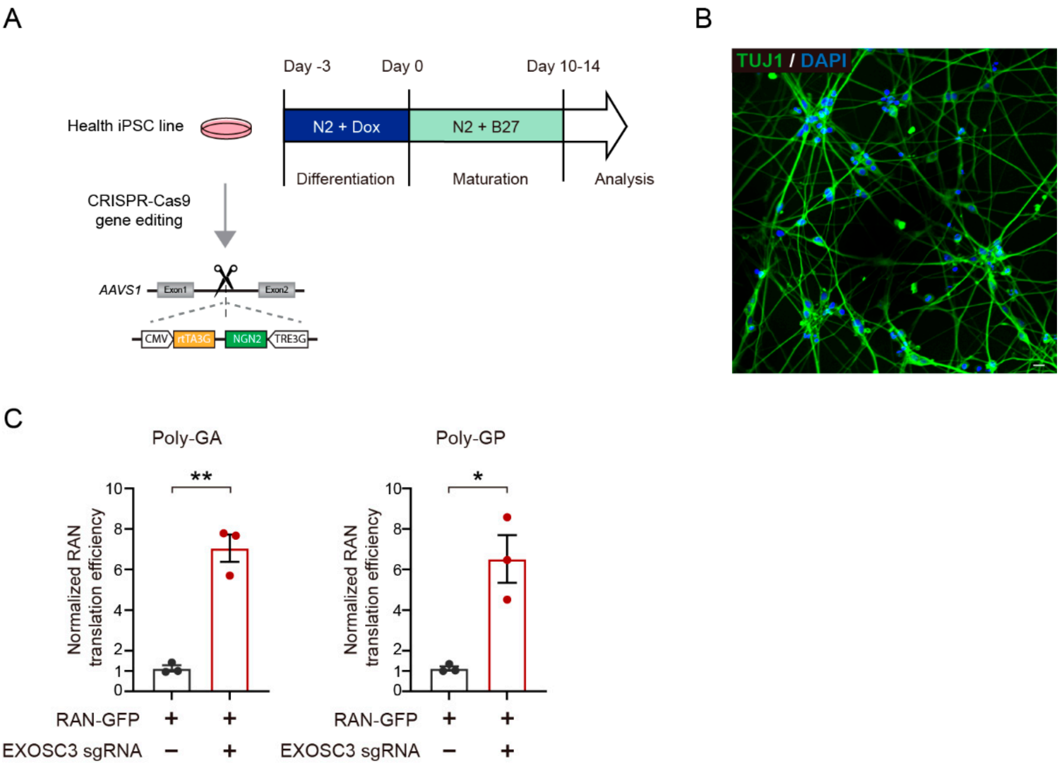

Figure S5. Establishment of i3N iPSCs and validation of differentiated i3Neurons.

A Overview of i3N iPSC generation and i3Neuron differentiation.

B Representative immunofluorescence images of day 10 (d10) differentiated i3Neurons showed positive staining for the neuronal marker TUJ1. Nuclei were visualized by DAPI (blue). Scale bar: 10 µm.

C Calculated RAN translation efficiencies for the production of poly-GA or poly-GP in RAN-GFP-expressing i3Neurons with or without EXOSC3 depletion (n = 3).

Data are presented as mean ± SEM. Statistical significance was determined using unpaired two-sided Student's t-test; *P < 0.05; **P < 0.01; ***P < 0.001; ns, not significant.
