# Supplementary material for: RAN Translation-Coupled Decay of the C9orf72 GGGGCC Repeat Transcript by the RNA Exosome Suppresses Dipeptide Repeat Production
Source: Int J Mol Sci. 2026 Aug 4;27(15):6986. doi: 10.3390/ijms27156986 (PMC13466912; doi:10.3390/ijms27156986)
Supplement: Supplementary file 1 [file ijms-27-06986-s001.zip › Table S1.pdf]

Table S1: Plasmid sequences

Plasmid	Sequence (5'-3')
RAN-GFP	TAACTAAGTAAGGATCCGTGTGTGTTTTTGTTTTTCCCACCCTCTCTCCCCACTACTTGCT<br>CTCACAGTACTCGCTGAGGGTGAACAAGAAAAGACCTGATAAAGATTAACCAGAAGAAAA<br>CAAGGAGGGAAACAACCGCAGCCTGTAGCAAGCTCTGGAACTCAGGAGTCGCGCGCTA<br>CCC**(GGGGCC)n**GGCGTCGACGTGAGCAAGGGCGAGGAGCTGTTCACCGGGGTGGTGC<br>CCATCCTGGTCGAGCTGGACGGCGACGTAAACGGCCACAAGTTCAGCGTGTCCGGCGA<br>GGGCGAGGGCGATGCCACCTACGGCAAGCTGACCCTGAAGTTCATCTGCACCACCGGC<br>AAGCTGCCCGTGCCCTGGCCCACCCTCGTGACCACCCTGACCTACGGCGTGCAGTGCTT<br>CAGCCGCTACCCCGACCACATGAAGCAGCACGACTTCTTCAAGTCCGCCATGCCCGAAG<br>GCTACGTCCAGGAGCGCACCATCTTCTTCAAGGACGACGGCAACTACAAGACCCGCGCC<br>GAGGTGAAGTTCGAGGGCGACACCCTGGTGAACCGCATCGAGCTGAAGGGCATCGACT<br>TCAAGGAGGACGGCAACATCCTGGGGCACAAGCTGGAGTACAACTACAACAGCCACAAC<br>GTCTATATCATGGCCGACAAGCAGAAGAACGGCATCAAGGTGAACTTCAAGATCCGCCA<br>CAACATCGAGGACGGCAGCGTGCAGCTCGCCGACCACTACCAGCAGAACACCCCCATC<br>GGCGACGGCCCCGTGCTGCTGCCCGACAACCACTACCTGAGCACCCAGTCCGCCCTGA<br>GCAAAGACCCCAACGAGAAGCGCGATCACATGGTCCTGCTGGAGTTCGTGACCGCCGC<br>CGGGATCACTCTCGGCATGGACGAGCTGTACAAGTAG
ATG-GFP	GCCACCATGGTGAGCAAGGGCGAGGAGCTGTTCACCGGGGTGGTGCCCATCCTGGTCG<br>AGCTGGACGGCGACGTAAACGGCCACAAGTTCAGCGTGTCCGGCGAGGGCGAGGGCGA<br>TGCCACCTACGGCAAGCTGACCCTGAAGTTCATCTGCACCACCGGCAAGCTGCCCGTGC<br>CCTGGCCCACCCTCGTGACCACCCTGACCTACGGCGTGCAGTGCTTCAGCCGCTACCCC<br>GACCACATGAAGCAGCACGACTTCTTCAAGTCCGCCATGCCCGAAGGCTACGTCCAGGA<br>GCGCACCATCTTCTTCAAGGACGACGGCAACTACAAGACCCGCGCCGAGGTGAAGTTCG<br>AGGGCGACACCCTGGTGAACCGCATCGAGCTGAAGGGCATCGACTTCAAGGAGGACGG<br>CAACATCCTGGGGCACAAGCTGGAGTACAACTACAACAGCCACAACGTCTATATCATGGC<br>CGACAAGCAGAAGAACGGCATCAAGGTGAACTTCAAGATCCGCCACAACATCGAGGACG<br>GCAGCGTGCAGCTCGCCGACCACTACCAGCAGAACACCCCCATCGGCGACGGCCCCGT<br>GCTGCTGCCCGACAACCACTACCTGAGCACCCAGTCCGCCCTGAGCAAAGACCCCAACG<br>AGAAGCGCGATCACATGGTCCTGCTGGAGTTCGTGACCGCCGCCGGGATCACTCTCGG<br>CATGGACGAGCTGTACAAGTAG
RAN-Flag	TAACTAAGTAAGGATCCGTGTGTGTTTTTGTTTTTCCCACCCTCTCTCCCCACTACTTGCT<br>CTCACAGTACTCGCTGAGGGTGAACAAGAAAAGACCTGATAAAGATTAACCAGAAGAAAA<br>CAAGGAGGGAAACAACCGCAGCCTGTAGCAAGCTCTGGAACTCAGGAGTCGCGCGCTA<br>CCC**(GGGGCC)n**GGCGTCGACGATTACAAGGATGACGATGACAAGTAG

ATG-Flag	GCCACCATGGTGAGCAAGGGCGAGGAGCTGTTCACCGGGGTGGTGCCCATCCTGGTCG<br>AGCTGGACGGCGACGTAAACGGCCACAAGTTCAGCGTGTCCGGCGAGGGCGAGGGCGA<br>TGCCACCTACGGCAAGCTGACCCTGAAGTTCATCTGCACCACCGGCAAGCTGCCCGTGC<br>CCTGGCCCACCCTCGTGACCACCCTGACCTACGGCGTGCAGTGCTTCAGCCGCTACCCC<br>GACCACATGAAGCAGCACGACTTCTTCAAGTCCGCCATGCCCGAAGGCTACGTCCAGGA<br>GCGCACCATCTTCTTCAAGGACGACGGCAACTACAAGACCCGCGCCGAGGTGAAGTTCG<br>AGGGCGACACCCTGGTGAACCGCATCGAGCTGAAGGGCATCGACTTCAAGGAGGACGG<br>CAACATCCTGGGGCACAAGCTGGAGTACAACTACAACAGCCACAACGTCTATATCATGGC<br>CGACAAGCAGAAGAACGGCATCAAGGTGAACTTCAAGATCCGCCACAACATCGAGGACG<br>GCAGCGTGCAGCTCGCCGACCACTACCAGCAGAACACCCCCATCGGCGACGGCCCCGT<br>GCTGCTGCCCGACAACCACTACCTGAGCACCCAGTCCGCCCTGAGCAAAGACCCCAACG<br>AGAAGCGCGATCACATGGTCCTGCTGGAGTTCGTGACCGCCGCCGGGATCACTCTCGG<br>CATGGACGAGCTGTACAAGGTCGACGATTACAAGGATGACGATGACAAGTAG
RAN-MS2	TAACTAAGTAAGGATCCGTGTGTGTTTTTGTTTTTCCCACCCTCTCTCCCCACTACTTGCT<br>CTCACAGTACTCGCTGAGGGTGAACAAGAAAAGACCTGATAAAGATTAACCAGAAGAAAA<br>CAAGGAGGGAAACAACCGCAGCCTGTAGCAAGCTCTGGAACTCAGGAGTCGCGCGCTA<br>CCC**(GGGGCC)n**GGCGTCGACGTGAGCAAGGGCGAGGAGCTGTTCACCGGGGTGGTGC<br>CCATCCTGGTCGAGCTGGACGGCGACGTAAACGGCCACAAGTTCAGCGTGTCCGGCGA<br>GGGCGAGGGCGATGCCACCTACGGCAAGCTGACCCTGAAGTTCATCTGCACCACCGGC<br>AAGCTGCCCGTGCCCTGGCCCACCCTCGTGACCACCCTGACCTACGGCGTGCAGTGCTT<br>CAGCCGCTACCCCGACCACATGAAGCAGCACGACTTCTTCAAGTCCGCCATGCCCGAAG<br>GCTACGTCCAGGAGCGCACCATCTTCTTCAAGGACGACGGCAACTACAAGACCCGCGCC<br>GAGGTGAAGTTCGAGGGCGACACCCTGGTGAACCGCATCGAGCTGAAGGGCATCGACT<br>TCAAGGAGGACGGCAACATCCTGGGGCACAAGCTGGAGTACAACTACAACAGCCACAAC<br>GTCTATATCATGGCCGACAAGCAGAAGAACGGCATCAAGGTGAACTTCAAGATCCGCCA<br>CAACATCGAGGACGGCAGCGTGCAGCTCGCCGACCACTACCAGCAGAACACCCCCATC<br>GGCGACGGCCCCGTGCTGCTGCCCGACAACCACTACCTGAGCACCCAGTCCGCCCTGA<br>GCAAAGACCCCAACGAGAAGCGCGATCACATGGTCCTGCTGGAGTTCGTGACCGCCGC<br>CGGGATCACTCTCGGCATGGACGAGCTGTACAAGTAAGCTAGCAACCTAGAAACGGGTG<br>GAGGATCACCCCACCCGACACTTCACAATCAAGGGGTACAATACACAAGGGTGGAGGAA<br>CACCCCACCCTCCAGACACATTACACAGAAATCCAATCAAACAGAAGCACCATCAGGGCT<br>TCTGCTACCAAATTTATCTCAAAAAACTACAACAAGGAATCACCATCAGGGATTCCCTGTG<br>CAATATACGTCAAACGAGGGCCACGACGGGAGGACGATCACGCCTCCCGAATATCGGCA<br>TGTCTGGCTTTCGTATTCAGTGCGTGGAGCATCAGCCCACGCAGCCAATCAGAGTCGAAT<br>ACAAGTCGAGTTTCGCGAAGAGCATCAGCCTTCGCGCCATTCTTACACAAACCACACTCT<br>CCCCTACAGGAACAGCATCAGCGTTCCTGCCCAGTACCCAACTCAAGAAAATTTATGTCC<br>CCATGCAGCATCAGCGCATGGGCCCCAAGAATACATCCCCAACAAAATCACATCCGAGC<br>ACCAACAGGGCTCGGAGTGTTGTTTCTTGTCCAACTGGACAAACCCTCCATGGACCATCA<br>GGCCATGGACTCTCACCAACAAGACAAAAACTACTCTTCTCGAAGCAGCATCAGCGCTTC<br>GAAACACTCGAGCATACATTGTGCCTATTTCTTGGGTGGACGATCACGCCACCCATGCTC

	TCACGAATTTCAAAACACGGACAAGGACGAGCACCACCAGGGCTCGTCGTTCCACGTCC<br>AATACGATTACTTACCTTTCGGGATGACGATCACGCATCCCGCAGCTACATCACTTCCAC<br>TCAGGACATTCAAGCATGCACGATCACGGCATGCTCCACAAGTCTCAACCACAGAAACTA<br>CCAAATGGGTTCAGCACCAGCGAACCCACTCCTACCTCAAACCTCTTCCCACAAAACTGG<br>CAAGCAGGATCACCGCTTGCCCATTCCAACATACCAAATCAAAAACAATTACTGGTACAG<br>CATCAGCGTACCAGCCCACATCTCTCACTACTATCAAAAACCAAACCGTTCAGCAACAGC<br>GAACGGTACACACGGAAAAATCAACTGGTTTACAAATACGAAAGACGATCACGCTTTCGT<br>CCAGCGCAAACTATTACGAAAAACATCCGACGGGAAGAGCAACAGCCTTCCCGCGGCGG<br>AAAACCTCACAAAAACACGACAAACGGATGCACGAACACGGCATCCGCCGACAACCCAC<br>AAACTTACAACCAGGCAAACGGTGCAGGATCACCGCACCGTACATCAAACACCTCAGATC<br>TCAT
