# Supplementary material for: RAN Translation-Coupled Decay of the C9orf72 GGGGCC Repeat Transcript by the RNA Exosome Suppresses Dipeptide Repeat Production
Source: Int J Mol Sci. 2026 Aug 4;27(15):6986. doi: 10.3390/ijms27156986 (PMC13466912; doi:10.3390/ijms27156986)
Supplement: Supplementary file 1 [file ijms-27-06986-s001.zip › Table S2.pdf]

Table S2: Primer sequences used for RT-qPCR

Primer	Sequence (5'-3')
EGFP-F	CTGGTCGAGCTGGACGGCGACG
EGFP-R	CACGAACTCCAGCAGGACCATGTGATCGC
EXOSC3-F	GTCTGTCGCGGCTGAATCTCTCG
EXOSC3-R	CTGTTCCGGCAGGAGCAGC
C9orf72-F	TGCTTACTGGAGAAGTGATTCCTGT
C9orf72-R	TGGTATCTGCTTCATCCAGCTTTT
GAPDH-F	GTCTCCTCTGACTTCAACAGCG
GAPDH-R	ACCACCCTGTTGCTGTAGCCAA
TUBB-F	GAGGAGATGACTCCTTCAACACC
TUBB-R	TGATGAGCTGCTCAGGGTGGAA
18SrRNA-F	ACCCGTTGAACCCCATTCGTGA
18SrRNA-R	GCCTCACTAAACCATCCAATCGG
